# Compensatory Response by Late Embryonic Tubular Epithelium to the Reduction in Pancreatic Progenitors

**DOI:** 10.1371/journal.pone.0142286

**Published:** 2015-11-05

**Authors:** Wataru Nishimura, Archana Kapoor, Ilham El Khattabi, Wanzhu Jin, Kazuki Yasuda, Susan Bonner-Weir, Arun Sharma

**Affiliations:** 1 Section of Islet Cell & Regenerative Biology, Joslin Diabetes Center, Boston, Massachusetts, United States of America; 2 Department of Medicine, Harvard Medical School, Boston, Massachusetts, United States of America; 3 Department of Metabolic Disorder, Diabetes Research Center, Research Institute, National Center for Global Health and Medicine, Tokyo, Japan; 4 Division of Anatomy, Bio-imaging and Neuro-cell Science, Jichi Univerisity, Tochigi, Japan; University of Minnesota Medical School, UNITED STATES

## Abstract

Early in pancreatic development, epithelial cells of pancreatic buds function as primary multipotent progenitor cells (1°MPC) that specify all three pancreatic cell lineages, i.e., endocrine, acinar and duct. Bipotent "Trunk" progenitors derived from 1°MPC are implicated in directly regulating the specification of endocrine progenitors. It is unclear if this specification process is initiated in the 1°MPC where some 1°MPC become competent for later specification of endocrine progenitors. Previously we reported that in *Pdx1* ^*tTA/+*^ *;tetO* ^*MafA*^ (bigenic) mice inducing expression of transcription factor MafA in Pdx1-expressing (Pdx1^+^) cells throughout embryonic development inhibited the proliferation and differentiation of 1°MPC cells, resulting in reduced pancreatic mass and endocrine cells by embryonic day (E) 17.5. Induction of the transgene only until E12.5 in Pdx1^+^ 1°MPC was sufficient for this inhibition of endocrine cells and pancreatic mass at E17.5. However, by birth (P0), as we now report, such bigenic pups had significantly increased pancreatic and endocrine volumes with endocrine clusters containing all pancreatic endocrine cell types. The increase in endocrine cells resulted from a higher proliferation of tubular epithelial cells expressing the progenitor marker Glut2 in E17.5 bigenic embryos and increased number of Neurog3-expressing cells at E19.5. A BrdU-labeling study demonstrated that inhibiting proliferation of 1°MPC by forced MafA-expression did not lead to retention of those progenitors in E17.5 tubular epithelium. Our data suggest that the forced MafA expression in the 1°MPC inhibits their competency to specify endocrine progenitors only until E17.5, and after that compensatory proliferation of tubular epithelium gives rise to a distinct pool of endocrine progenitors. Thus, these bigenic mice provide a novel way to characterize the competency of 1°MPC for their ability to specify endocrine progenitors, a critical limitation in our understanding of endocrine differentiation.

## Introduction

Early in pancreatic development, epithelial cells of pancreatic buds function as primary multipotent progenitor cells (1°MPC) and give rise to all three pancreatic cell lineages i.e, endocrine, acinar and duct [1–3]. Subsequent expansion and remodeling of epithelium results in compartmentalization of these cells into 1) bipotent "Trunk" cells that are considered to differentiate into endocrine and ductal cells and 2) secondary MPC (2°MPC) "Tip" cells that initially specify all three pancreatic cell types and become later restricted to only acinar cells [4]. Similarly, towards the end of gestation and after birth the fate of bipotent Trunk epithelial cells became increasingly restricted to ductal cells. The size of the 1°MPC pool is thought to determine the pancreatic size, with the pancreas lacking a compensatory response for restoring lost pancreatic cell types after a reduction in progenitor pool [5]. This schema suggests that the specification of endocrine, acinar and ductal progenitor fate was committed in the 1°MPCs, and in case of endocrine cells, much before the increase in Neurog3^+^ endocrine progenitors during the secondary transition. However, it is unclear whether all 1°MPCs acquire endocrine competence, how they contribute to specification of endocrine progenitors from the "Trunk" epithelium, and whether they regulate specification of endocrine progenitors throughout the embryonic development or only during secondary transition. A better understanding of these early steps of endocrine differentiation should enhance our ability to convert pancreatic progenitors into endocrine progenitors and increase the efficiency of β-cell generation from stem/progenitor cells.

The roles of embryonic Trunk/tubular epithelium and postnatal ductal cells in the neogenesis of insulin-producing cells have been studied using multiple approaches including lineage tracing [4, 6–16]. Early in development, Hnf1β^+^ cells in embryonic ductal epithelium function as precursors of all three pancreatic lineages, but after E16.5 these cells do not differentiate into acinar or endocrine cells [8]. Up until P1, Sox9^+^ cells in tubular epithelium can differentiate into both endocrine and acinar cells, but they lose this differentiation capacity shortly after birth [11, 12]. Hence, it is generally accepted that the early embryonic tubular epithelial cells have a high differentiation capacity, but with increasing gestational age their ability to differentiate into endocrine cells is lost or reduced. Since the 1°MPC pool may dictate the final size of the pancreas [5], possibly by controlling the capacity of embryonic tubular epithelium to proliferate and differentiate into all pancreatic cell types, it is likely that the reduced differentiation potential of late embryonic tubular epithelium is also regulated at the level of 1°MPC. These observations suggest that the endocrine competency of 1°MPC may regulate differentiation of endocrine cells during both the secondary transition and late-gestational period.

We previously generated transgenic mice *Pdx1* ^*tTA/+*^ *;tetO* ^*MafA*^ (bigenic) expressing the insulin gene transcription MafA in Pdx1^+^ cells [17]. Expression of *MafA* transgene (MafA^Myc^) in Pdx1^+^ cells throughout embryonic development prevented the proliferation and differentiation of those cells. By E17.5, the bigenic pancreas was significantly smaller and contained only a few endocrine cells. The relative endocrine volume of E17.5 bigenic pancreas was less than 5% that of littermate controls [17]. Importantly, the expression of *MafA* ^*Myc*^ in Pdx1^+^ cells until E12.5 (in 1°MPC) was sufficient to recapitulate this phenotype, supporting a role for 1°MPC in regulating endocrine differentiation during secondary transition. Thus, *Pdx1* ^*tTA/+*^ *;tetO* ^*MafA*^ mice provide an opportunity to examine the role of 1°MPC in endocrine differentiation throughout embryonic development as well as to evaluate the endocrine differentiation potential of late-embryonic tubular epithelium.

Here we report that permitting bigenic pups to reach term resulted in a significant increase in both mass and number of endocrine cells between E17.5 and P0, which was not due to enhanced proliferation of rare endocrine cells present in E17.5 bigenic pancreas but rather to enhanced proliferation of tubular epithelial cells and increased numbers of Neurog3-expressing endocrine progenitors. We show that only the tubular epithelium in the E17.5 bigenic but not control pancreas expressed the progenitor marker GLUT2 and this increase in progenitors occurred without preferential retention of BrdU-labeled 1°MPC in tubular epithelium. Thus, MafA expression in the 1°MPC of bigenic pancreas inhibits their competency to specify pancreatic and endocrine cells only until E17.5, and the endocrine progenitors specified after E17.5 are either not dependent on 1°MPC competency or a distinct progenitor population is induced due to compensatory proliferation of tubular epithelium. We suggest that examining this role of 1°MPC in regulating specification of endocrine progenitors before and after E17.5 may provide alternate pathways to generate β-cells from stem cells.

## Materials and Methods

### Ethics Statement

All animal experiments were approved by the Joslin Institutional Animal Care and Use Committee.

### Generation of transgenic mice

Transgenic *tetO* ^*MafA*^ mice [17] were crossed with *Pdx1* ^*tTA/+*^ mice [18] (purchased from Jackson Laboratory, Bar Harbor, ME) to generate *Pdx1* ^*tTA/+*^ *;tetO* ^*MafA*^ transgenic mice as described previously [17]. If not specifically indicated, wild type and *tetO* ^*MafA*^ littermates were used as controls in this study.

### Animals

The day of vaginal-plug discovery was designated as E (embryonic day) 0.5. The date of birth was designated as P (postnatal day) 0. Studies were performed without providing Doxycycline which results in *MafA* ^*myc*^ transgene expression being induced in cells expressing Pdx1. Blood glucose values were measured on blood from tail snip using One-Touch glucometer (Life Scan, Milpitas, CA). For BrdU study, pregnant dams were injected with 100mg/kg BrdU in PBS on gestational days 10.5 and 11.5. Animals were sacrificed for excision of the pancreas at E17.5, E19.5, P0 or P1. Pancreas was fixed in 4% paraformaldehyde and embedded in paraffin for immunohistochemical studies.

### Immunohistochemistry and quantification

Immunostaining analyses of at least 3 animals of each genotype were performed on 4 μm paraffin sections as described previously [19]. The primary antibodies used were: guinea pig anti-insulin (Linco, Billerica, MA); guinea pig anti-glucagon (Linco); rabbit anti-glucagon (provided by Dr. M Appel); rabbit anti-somatostatin (Chemicon International, Billerica, MA); rabbit anti-ghrelin (Phoenix Pharmaceuticals, Belmont, CA); rabbit anti-pancreatic polypeptide (Linco); mouse anti-Nkx2.2 (Developmental Studies Hybridoma Bank, Iowa City, IA); rabbit anti-Nkx6.1 (provided by Dr. P Serup); mouse anti-Neurog3 (provided by Drs. Serup and O Madsen, Beta Cell Biology Consortium); rabbit anti-Pax6 (Covance, Princeton, NJ); rabbit anti-Pdx1 (provided by Dr. J Slack); mouse anti-Isl1 (Developmental Studies Hybridoma Bank, Iowa City, IA); mouse anti-Hb9 (provided by Dr. S. Pfaff); mouse anti-Ki67 (BD Pharmingen, San Jose, CA); rabbit anti-Glut2 (Chemicon International); mouse anti-E-cadherin (BD Transduction Laboratories, San Jose, CA); rabbit anti-Myc (Cell Signaling Technology, Danvers, MA); rat anti-BrdU (Abcam, Cambridge, MA). Biotinylated-DBA lectin (Vector, Burlingame, CA) was used to identify duct cells. For amplification biotinylated anti-rabbit or anti-mouse antibodies (Jackson ImmunoResearch, West Grove, PA) were used at 1:400 dilution followed by streptavidin-conjugated Alexafluor 488 (1:400) (Molecular Probes, Eugene, OR). For amplifying the Neurog3 staining, TSA kit from Perkin Elmer was used. Secondary antibodies were Texas red-conjugated anti-rabbit, anti-mouse or anti-guinea pig IgG (Jackson ImmunoResearch). DAPI mounting medium (Vector Lab) was used to label the nuclei. Immunofluorescent images were obtained using Zeiss LSM410 (Zeiss, Thornwood, NY) in confocal mode. Acquired images were identically processed using Adobe Photoshop CS2.

Ki67^+^ expression was quantified in 261–528 DBA^+^ and 68–280 insulin^+^ cells from at least 20 random microscopic fields of at least 3 embryos of each genotype using NIH Image J software. The cell number counted for insulin was low since insulin expression was inhibited in the E17.5 bigenic pancreases. Hormone^+^ area (insulin^+^ or glucagon^+^ area) / area of pancreatic tissue (as marked by DAPI) was measured on every 10^th^ section for each pancreas of at least three embryos or neonates for each genotype using BIOREVO BZ-9000 microscope; quantification was performed by BZ-H1C dynamic cell count (Keyence, Osaka, Japan). At least three E19.5 embryos were used to quantify Neurog3 cells within the DBA^+^ pancreatic cells. Five sections at least 10μm apart were stained with DBA, Neurog3 and DAPI. To normalize the number of Neurog3-expressing cells, the total pancreatic tissue and DBA^+^ areas were quantified using the ImageJ software. A total of 400–500 Neurog3 cells were quantified per embryo. Data are presented as mean ± s.e.m.; statistical significance was determined using the two-tailed unpaired Student's *t* test.

## Results

### E17.5 tubular epithelium of Pdx1^tTA/+^;tetO^MafA^ mice lack MafA^Myc^ transgene expression but some cells still expresses transcription factors involved in endocrine differentiation

Previously using bigenic *Pdx1* ^*tTA/+*^ *;tetO* ^*MafA*^ mice (DOX-OFF), in which the absence of DOX triggered the *MafA* ^*Myc*^ transgene (*tetO* ^*MafA*^) expression in Pdx1^+^ cells, we demonstrated that MafA misexpression in Pdx1^+^ primary multipotent pancreatic progenitors (1°MPC) reduced the pancreatic and endocrine mass by E17.5 and that its misexpression only until E12.5 was sufficient to recapitulate the E17.5 phenotype [17]. In bigenic mice at E12.5, Pdx1 and MafA^Myc^ were co-expressed in 1°MPCs, but by E15.5, cells expressing Pdx1 and MafA^Myc^ were significantly reduced in number [17]. These observations are consistent with the high level of *Pdx1* normally expressed in the pancreatic 1°MPCs until E12.5, after which high Pdx1 expression (Pdx1^Hi^) is reduced in the pancreatic epithelium but is turned-on in newly forming β-cells after E13.5 [19–21].

**Fig 1:**
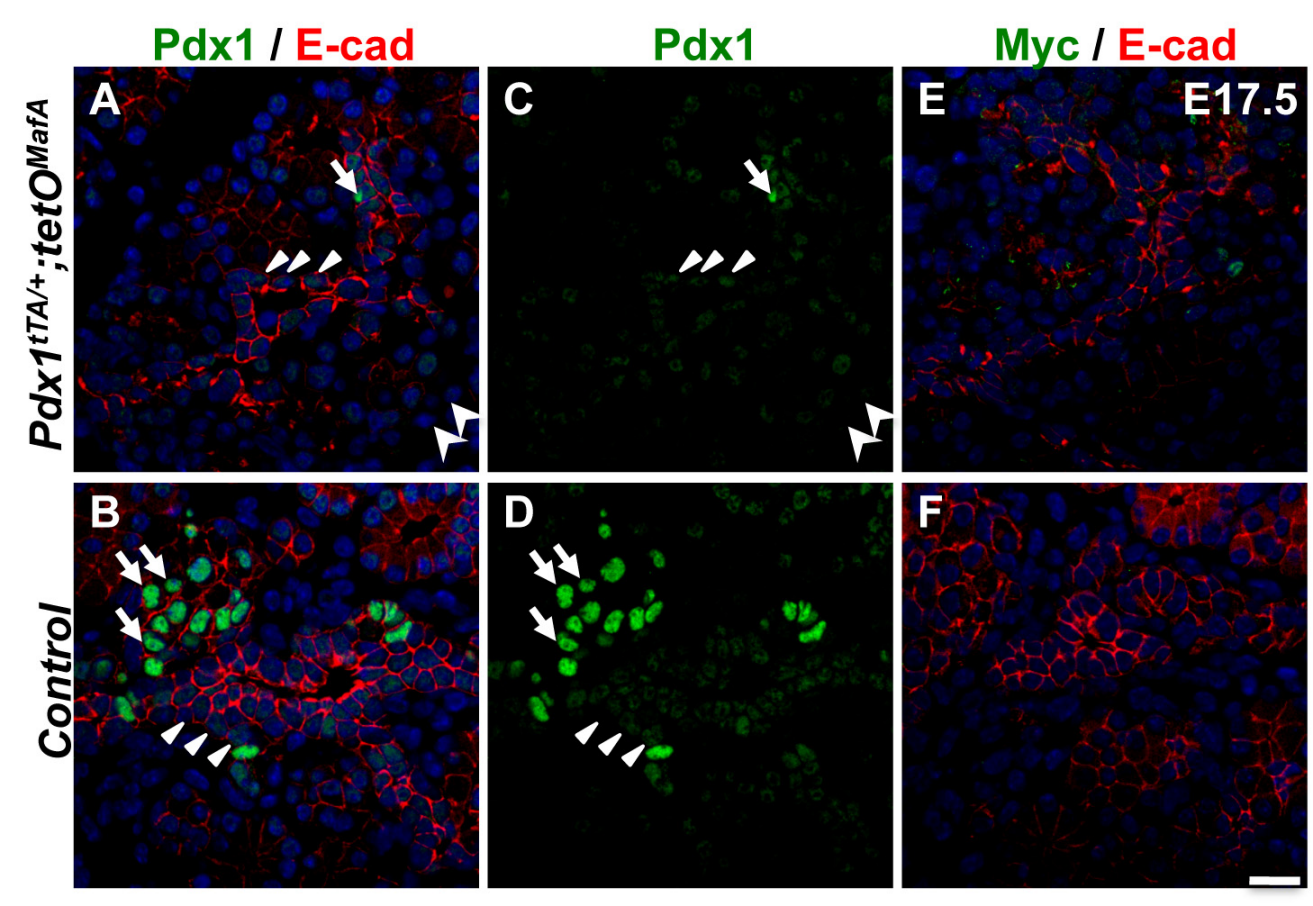
E17.5 tubular epithelium of bigenic pancreas expresses neither Pdx1^Hi^ nor MafA^Myc^. E17.5 bigenic and control pancreases were stained for E-cadherin (red, **ABEF**), Pdx1 (green, **ABCD**) and Myc (green, **EF**). Pdx1^Hi^, Pdx1^Lo^ and Pdx1^-^ cells are marked by arrows, triangles and arrowheads, respectively. In controls, tubular epithelial cells have Pdx1^Lo^ expression while Pdx1^Hi^ expression was only seen in endocrine cells. In bigenic pancreas only occasional Pdx1^Hi^ cells and MafA^Myc^ cells were seen indicating that Pdx1^Lo^ expression was not sufficient for *Pdx1* ^*tTA*^-dependent induction of MafA^Myc^ expression (green, **EF**). DAPI (blue). Bar: 20 μm.

**Fig 2:**
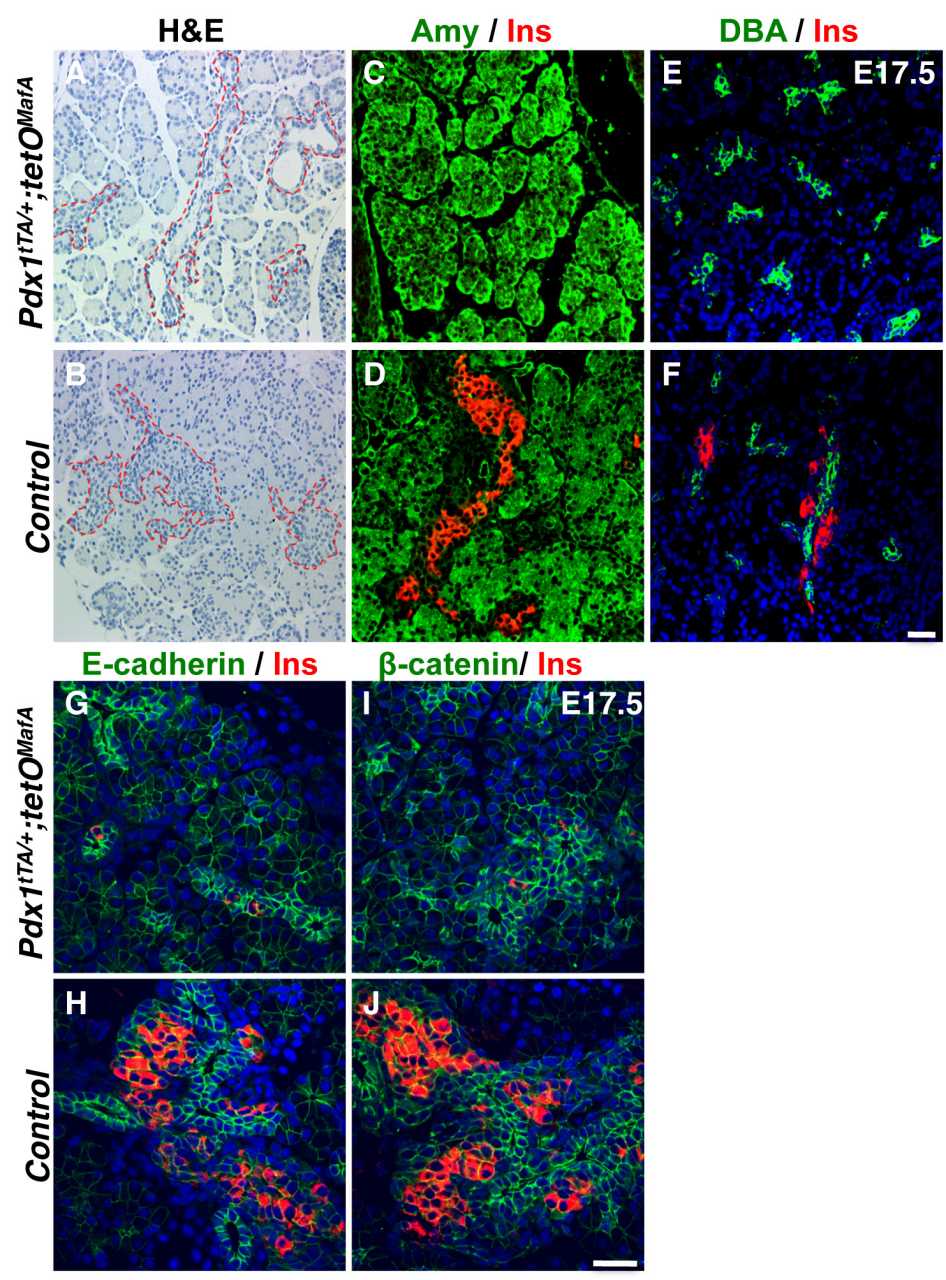
Normal-appearing acinar and tubular epithelial cells in E17.5 bigenic pancreas. H& E stained pancreatic sections from the E17.5 control and bigenic *Pdx1* ^*tTA/+*^ *;tetO* ^*MafA*^ mice (**AB**) show tubular epithelium and surrounding endocrine area (dashed line) with a lack of endocrine cells in the bigenic pancreas. Amylase (green, **CD**), insulin (red **C-J**), E-cadherin (green, **GH**), β-catenin (green, **IJ**), and DBA lectin (green **EF**) staining show a reduction in insulin^+^ cells in the bigenic pancreas but normal appearance of acinar and tubular epithelium. DAPI (blue). Bars: 50 μm.

Since cells in the embryonic Trunk/tubular epithelium are primarily responsible for the specification of pancreatic endocrine cells, we first examined whether the inhibitory effects of MafA^Myc^ expression on endocrine differentiation of 1°MPCs persisted in E17.5 pancreas. In controls, Pdx1^Hi^-expressing cells had low E-cadherin expression at E17.5 (Fig 1), while the high E-cadherin-expressing tubular epithelial cells showed Pdx1^Lo^ expression (Fig 1B and 1D). In the bigenic pancreas Pdx1^Hi^-expressing cells were dramatically reduced in number, with most cells in the tubular epithelium having Pdx1^Lo^ expression and lacking MafA^Myc^ expression (Fig 1A, 1C and 1E). The E17.5 tubular epithelium in the bigenic pancreas, like E15.5 epithelium [17], did not express MafA^Myc^. These observations that only the cells having higher Pdx1 expression were capable of inducing MafA^Myc^ expression (Fig 1E) are consistent with the 1°MPCs being the only progenitor population during the pancreatic development expressing the transgene MafA^Myc^ [17] and, therefore, likely responsible for the MafA^Myc^-dependent reduced number of endocrine progenitors and hormone-producing cells. The expression of *MafA* ^*Myc*^ in the 1°MPC did not alter the morphology of E17.5 tubular epithelial cells or their expression of E-cadherin and β-catenin nor that of the acinar cells (Fig 2) or DBA^+^ cells. The main difference in the bigenic mice was the lack of endocrine clusters surrounding the embryonic tubules.

**Fig 3:**
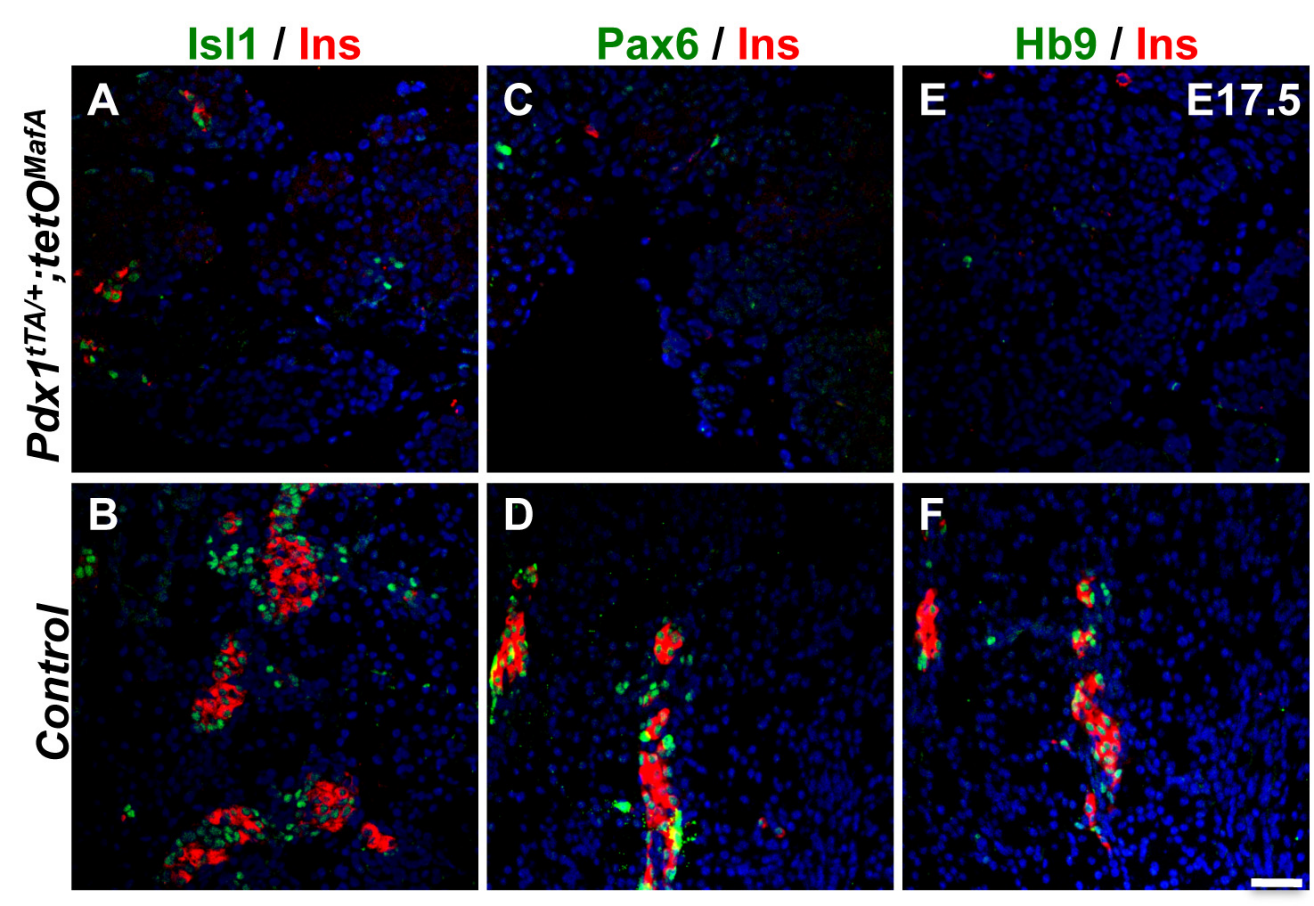
Endocrine differentiation of pancreas is impaired in *Pdx1* ^*tTA/+*^ *;tetO* ^*MafA*^ pancreas. At E17.5 Isl1 (green, **AB**), Pax6 (green, **CD**) and Hb9 (green, **EF**), transcription factors implicated in endocrine differentiation and maturation, have severely reduced expression in *Pdx1* ^*tTA/+*^ *;tetO* ^*MafA*^ pancreas (**ACE**) compared to *tetO* ^*MafA*^ pancreas (**BDF**). This finding is consistent with their reduced number of insulin^+^ cells (red, **A-F**), and suggests that misexpression of the MafA transgene inhibits the entire endocrine differentiation program. DAPI (blue). Bar: 50 μm.

To examine whether the effect of MafA^Myc^ expression in the Pdx1^+^ 1°MPC was temporally limited to the peak of endocrine differentiation (secondary transition), we examined the expression of genes regulating endocrine differentiation in bigenic pancreas. At E17.5, cells expressing the endocrine transcription factors Isl1, Pax6 and Hb9, as well as hormone-expressing cells, were dramatically reduced in number (Fig 3). A few insulin, Nkx2.2, Nkx6.1 and Neurog3 expressing cells were observed near or within the embryonic tubular structures (dashed lines in S1 Fig) in the bigenic pancreas. These results suggested that E17.5 tubular epithelial cells of bigenic mice retain the potential to differentiate into endocrine cells.

### A significant increase in endocrine cells and pancreatic size in P0 bigenic pancreas

**Fig 4:**
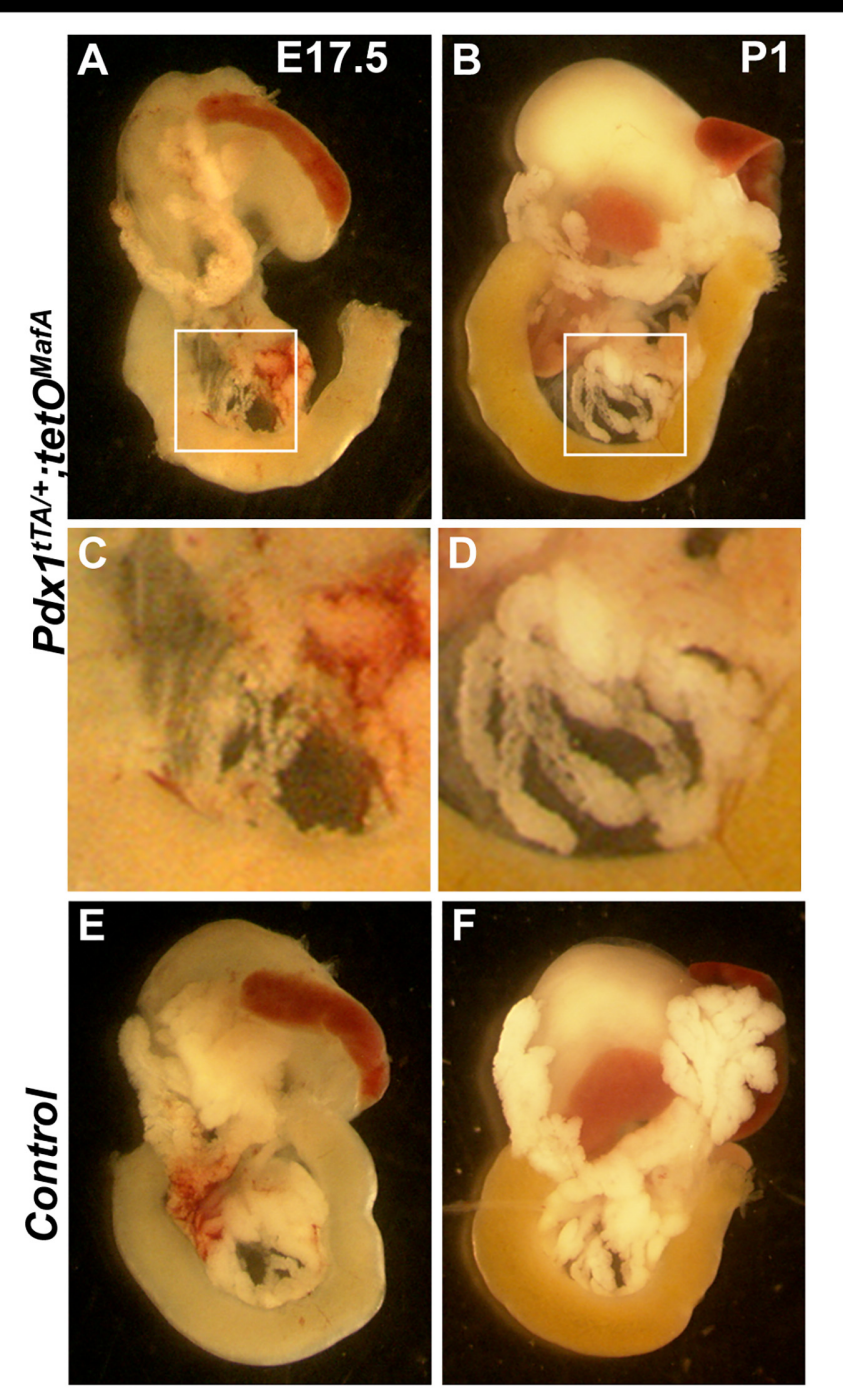
Pancreas of *Pdx1* ^*tTA/+*^ *;tetO* ^*MafA*^ embryos show dramatic growth from E17.5 to P0. Abdominal viscera were dissected from indicated genotypes at E17.5 and P0 (**A-F**). Continuous expression of MafA^Myc^ transgene in Pdx1^+^ cells until E17.5 resulted in dramatic reduction in pancreatic mass (**A**). However, in P0 bigenic neonates there is significant growth of pancreatic tissue along the duct structures compared to E17.5 bigenic embryos as shown in B and D, which show higher magnification of the boxed areas in **A** and **C**.

**Fig 5:**
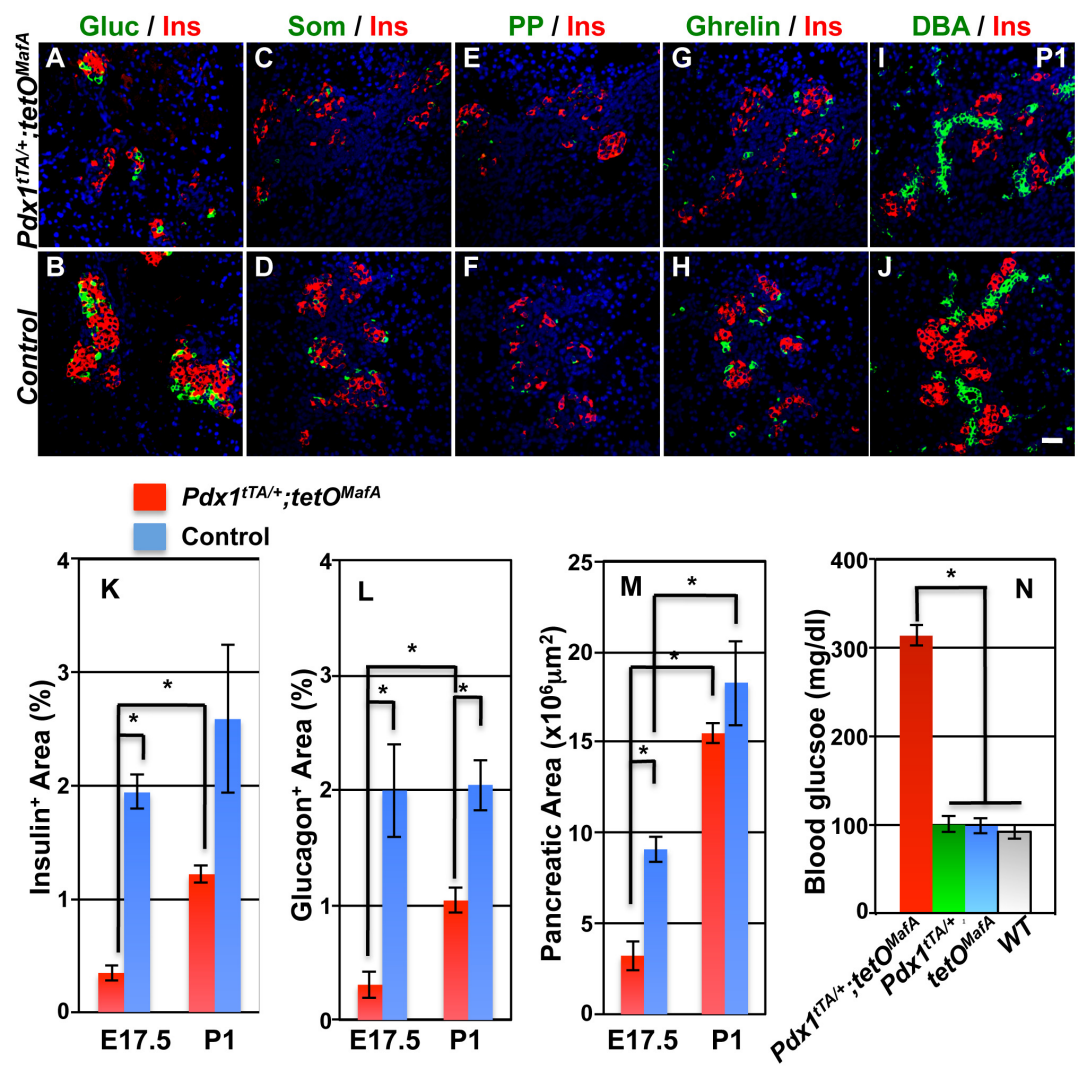
P1 transgenic pancreases contain significantly larger endocrine clusters compared to E17.5. At P1 bigenic islets had relatively normal organization as compared to controls for expression of glucagon (green, **AB**, red, **EF**), somatostatin (green, **CD**), pancreatic polypeptide (green, **EF**), ghrelin (green, **GH**) and insulin (red, **A-D**, **G-J**). DBA-expressing branching ducts were observed in both neonates (green, **IJ**). DAPI (blue). Bar: 50 μm. Quantification of these data show increases in the insulin^+^ and glucagon^+^ area of bigenic pancreas from E17.5 to P1 compared to controls (**KL**). Total pancreatic area in *Pdx1* ^*tTA/+*^ *;tetO* ^*MafA*^ also shows enhanced growth (**M**). Blood glucose levels from each group of neonates (n = 8 for each group) show that bigenic neonates were significantly hyperglycemic at P1 (**N**). Mean ± s.e.m.

We examined whether the remaining E17.5 tubular epithelial cells could function as bipotent/multipotent progenitors by allowing bigenic embryos to reach term. Pancreatic size in bigenic mice significantly increased between E17.5 and P1 (Fig 4A and 4C) but remained smaller than the controls (Fig 4E and 4F). Bigenic neonates showed a rapid growth in the pancreas mainly along the embryonic ductal structures (Fig 4B and 4D). P1 pancreatic sections from bigenic pups stained for endocrine hormones and ductal marker DBA showed endocrine cell clusters containing all endocrine cell types (Fig 5A–5J) with a greater increase in the relative acinar and endocrine cell volumes between E17.5 to P1 in bigenics than in controls. From E17.5 to P1, the insulin^+^ area in *Pdx1* ^*tTA/+*^ *;tetO* ^*MafA*^ mice increased 3.8-fold (p<0.01) compared to 1.3-fold in controls (Fig 5K), glucagon^+^ area increased 3.0-fold (p = 0.02) and 1.1-fold in bigenics and controls, respectively (Fig 5L), and total pancreatic area increased 4.8-fold (p<0.01) and 2.0-fold (p<0.01), respectively (Fig 5M). Although a significant growth was seen in the bigenic pancreas between E17.5 and P1, bigenic pancreas was still dramatically smaller than control (Fig 4). Additionally the partial recovery of endocrine cells in P1 bigenic pancreas was insufficient to prevent the development of hyperglycemia, with P1 bigenic pups having significantly higher blood glucose values (313.9±11.2 mg/dl) than the monogenic (*Pdx1* ^*tTA/+*^ or *tetO* ^*MafA*^) or wild type animals (99.9±9.1, 98.5±8.2 and 92.0±8.0 mg/dl, respectively) (Fig 5N).

### Increased proliferation of tubular epithelial cells but not of insulin^+^ cells in Pdx1^tTA/+^;tetO^MafA^ embryos

**Fig 6:**
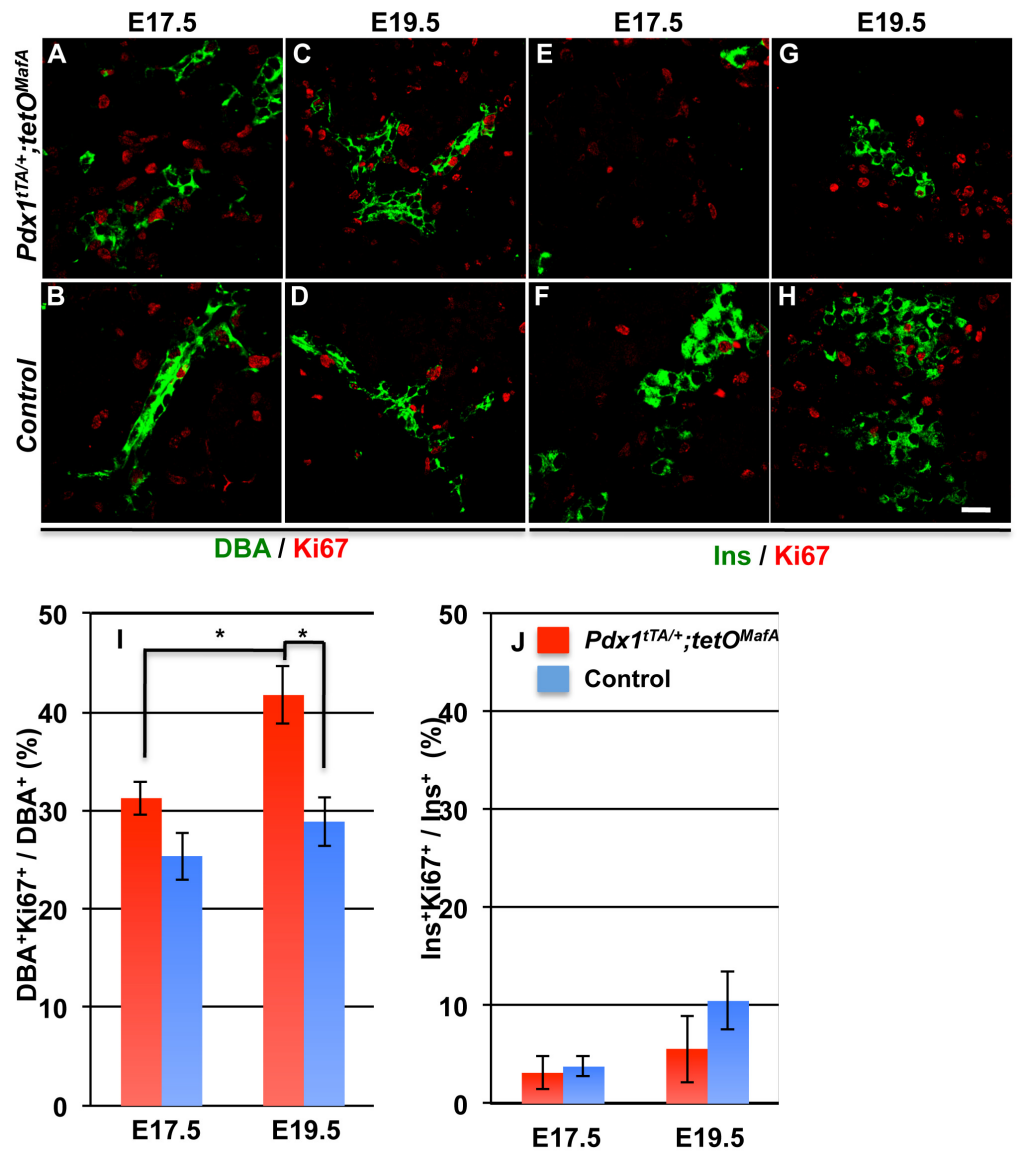
Proliferation of DBA^+^ epithelial tubules and not insulin^+^ cells contributes to increased number of insulin^+^ cells in *Pdx1* ^*tTA/+*^ *;tetO* ^*MafA*^ pancreas at E19.5 and later. Bigenic pancreas at both E17.5 and E19.5 showed proliferation (Ki67, red) of DBA^+^ (green, **A-D**) and insulin^+^ (green, **E-H**) cells. Quantification of the proportion of DBA^+^ cells or insulin^+^ cells that were Ki67^+^ showed significantly increased proliferation of DBA^+^ cells between E17.5 and E19.5 (**I**) in bigenic but not control pancreas whereas the proportion of insulin^+^ cells that were Ki67^+^ (**J**) at E19.5 compared to that at E17.5 increased in controls but not bigenic. The bigenic had significantly more replicating DBA^+^ cells at E19.5 than controls. N = 3 Mean ± s.e.m. Bar: 20 μm.

Endocrine cells formed during second transition remain quiescent until towards the end of gestation when they start to proliferate. In case of β-cells, this proliferation requires Pdx1 expression [22]. To discriminate between the possibilities that the increased qqβ-cells seen at birth were due to proliferation of the few endocrine cells present at E17.5 or from neogenesis or newly differentiated cells, we examined the co-expression of proliferation marker (Ki67) with DBA^+^ and insulin^+^ cells at E17.5 and E19.5 (Fig 6). At E17.5, 31.2±1.7% of DBA^+^ cells were Ki67^+^ in the bigenics while only 25.4±2.5% in controls. At E19.5, the proportion of DBA^+^ cells expressing Ki67 in *Pdx1* ^*tTA/+*^ *;tetO* ^*MafA*^ bigenics (41.7±2.9%, p = 0.02) was significantly increased than in controls (28.9±2.5%) (Fig 6A–6D and 6I). As shown previously [17], at E17.5 larger clusters of insulin^+^ cells were present in controls, but only occasional single insulin^+^ cells were seen in bigenic pancreas (Fig 6E and 6F). However by E19.5, we detected small clusters of insulin^+^ cells in the bigenic pancreas and a further increased size of insulin^+^ clusters in controls (Fig 6E–6H). At E17.5, the proportion of insulin^+^Ki67^+^ cells was comparable in *Pdx1* ^*tTA/+*^ *;tetO* ^*MafA*^ (3.1±1.6%) and control embryos (3.8±1.0%). At E19.5, the proliferation of insulin^+^ cells in bigenic (5.6±3.4%) did not differ from that at E17.5, while in controls insulin^+^ Ki67^+^ cells showed a tendency to increase (10.5±3.0%, p = 0.09) compared to E17.5 (3.8±1.0%). Thus at E19.5, embryonic tubular epithelial cells had greater proliferation in bigenic than in control pancreas, without significant changes in proliferation of insulin^+^ cells. These data support that the increased number of insulin^+^ cells seen in the bigenic pancreas at E19.5 and P1 (Figs 5 and 6) results from neogenesis, via proliferation and subsequent differentiation of DBA^+^ tubular cells, and not from increased proliferation of the few insulin^+^ cells present at E17.5.

### Tubular epithelial cells from Pdx1^tTA/+^;tetO^MafA^ embryos retain expression of a progenitor marker

**Fig 7:**
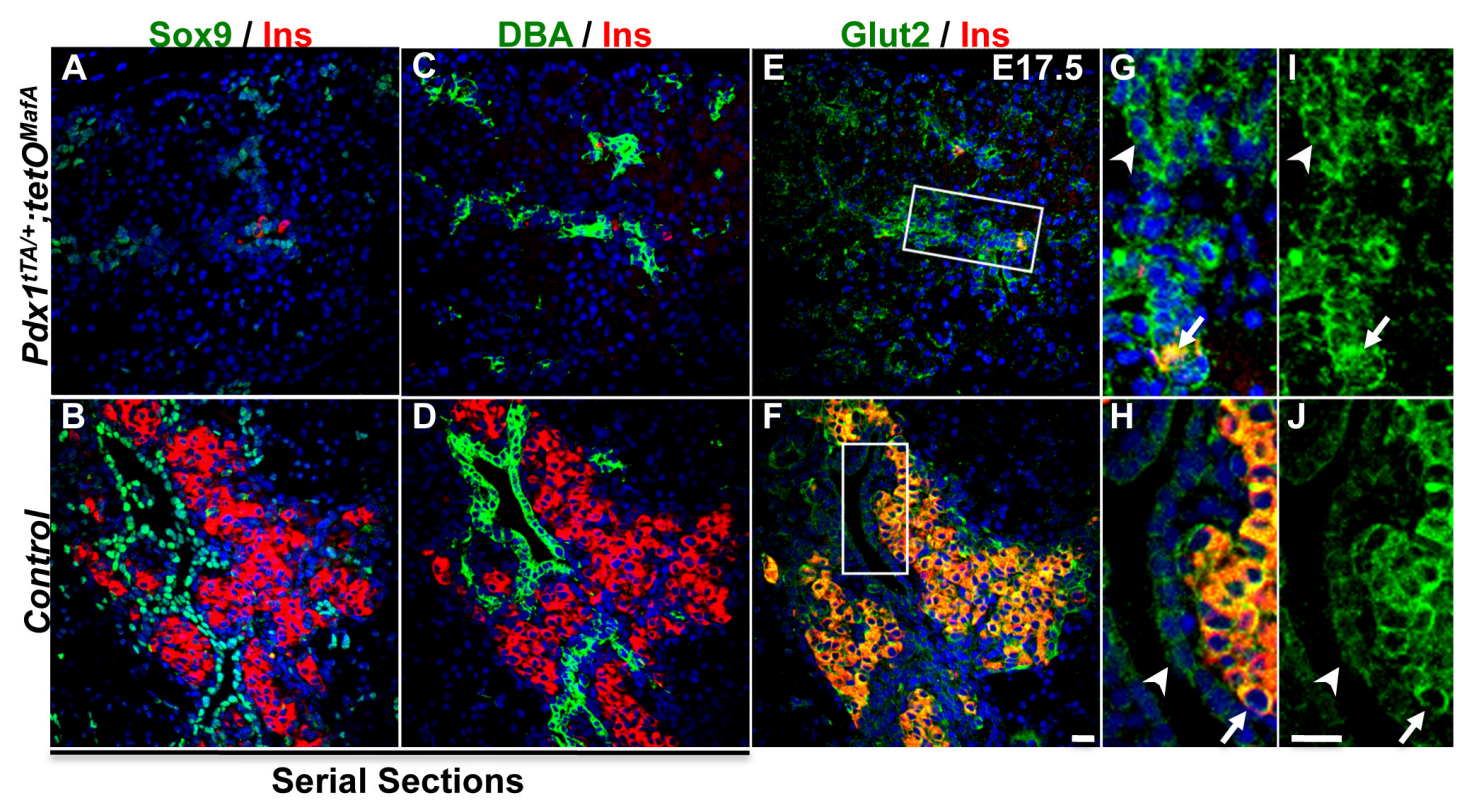
Tubular epithelial cells of bigenic pancreas express Sox9 and GLUT2 at E17.5. At E17.5 both control and bigenic tubular epithelial cells express Sox9. Sox9 (green **AB**); DBA (green, **CD**); GLUT2 (green, **EF**); Insulin (red); DAPI (blue). The boxed areas in E and F are enlarged (**GH**: merged channels, **IJ**: green channel showing GLUT2 expression). Higher GLUT2 staining intensity is seen in the bigenic tubular epithelial cells than in the controls. In bigenic pancreas GLUT2 staining intensity is comparable in insulin^+^ (marked by arrows) and insulin^-^ tubular epithelial cells (marked by arrowheads) whereas in control pancreas GLUT2 staining intensity is reduced in tubular epithelial cells than islets. Bar: 20 μm.

The expression of progenitor markers Sox9 and GLUT2 were examined to evaluate the enhanced neogeneic capacity of E17.5 DBA^+^ tubular cells in bigenic pancreas. Sox9 was expressed in the DBA^+^ tubular epithelial cells of both bigenic and control pancreas (Fig 7A–7D). Early in pancreatic development, GLUT2 expression marks the multipotent progenitors [23]. However, by E17 when the fate of tubular epithelial cells is becoming restricted to ducts, most of these cells lack Glut2 expression, except those that co-express insulin [23, 24]. Consistent with these published results, in E17.5 control pancreas GLUT2 expression in tubular epithelium was significantly reduced compared to the high GLUT2 expression in insulin^+^ cells (Fig 7F, 7H and 7J). However, Glut2 expression in DBA^+^ cells in *Pdx1* ^*tTA/+*^ *;tetO* ^*MafA*^ pancreas was higher than in controls, and comparable to that in the rare insulin^+^ cells seen in these bigenic pancreases (Fig 7E, 7G and 7I). This higher expression of Glut2 in DBA^+^ tubular epithelium of E17.5 bigenic pancreas suggests that these cells retain the progenitor potential to proliferate and differentiate into other pancreatic cell types.

### Increased progenitor marker expression in E17.5 tubular epithelium did not results from enhanced retention of 1°MPC

**Fig 8:**
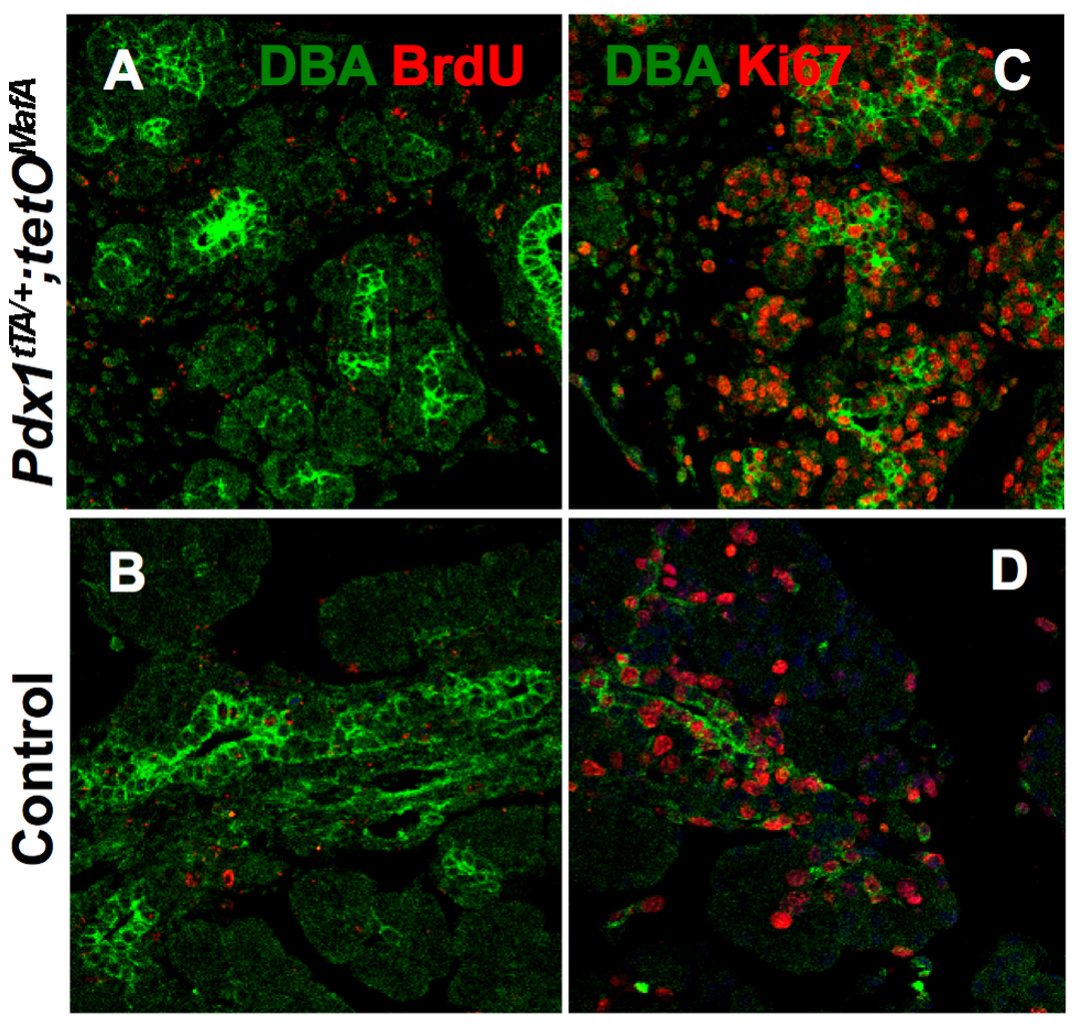
Retention of BrdU-labeled 1°MPC is not enhanced in E17.5 bigenic tubular epithelium. Images of E17.5 pancreases from bigenic and control pups from pregnant mothers receiving BrdU injections on both gestational days10.5 and 11.5, stained for BrdU (red, **AB**) and Ki67 (red, **CD**) with DBA (green). At this stage, only a few BrdU^+^ label-retaining cells remain in either E17.5 bigenic and control pancreas but many cells, including DBA^+^ tubular epithelium, are proliferating.

Expression of MafA^myc^ expression in 1°MPC prevented their proliferation and differentiation into endocrine cells. It is possible that some of the undifferentiated 1°MPC may remain in the tubular epithelium and when the repressive effects of MafA^myc^ expression is lost/removed, these progenitors regain their ability to differentiate into endocrine cells. Hence, we examined whether the enhanced progenitor potential of E17.5 bigenic tubular epithelium resulted from retention of 1°MPC. Pregnant dams were injected with BrdU on both gestational days 10.5 and 11.5 and were sacrificed on gestational day 17.5; the embryos were collected for genotyping and pancreatic staining. Sections were stained for DBA and BrdU (Fig 8A and 8B), as well as DBA and Ki67 (Fig 8C and 8D) to monitor proliferation of tubular epithelium and the presence of BrdU labeled 1°MPC. Results showed the expected increase in the proliferation of tubular epithelium at this stage of development in both bigenic and control pancreas. Furthermore, only rare tubular epithelial cells showed staining for BrdU. The lack of any increase in the BrdU-retaining cells in the bigenic tubular epithelial cells compared to those of controls demonstrate that the observed increase in pancreatic and endocrine cells between E17.5 and P1 in bigenic animals did not result from the 1°MPC that were retained in the tubular epithelium.

### E19.5 bigenic pancreas partially compensated for impaired induction of endocrine differentiation

**Fig 9:**
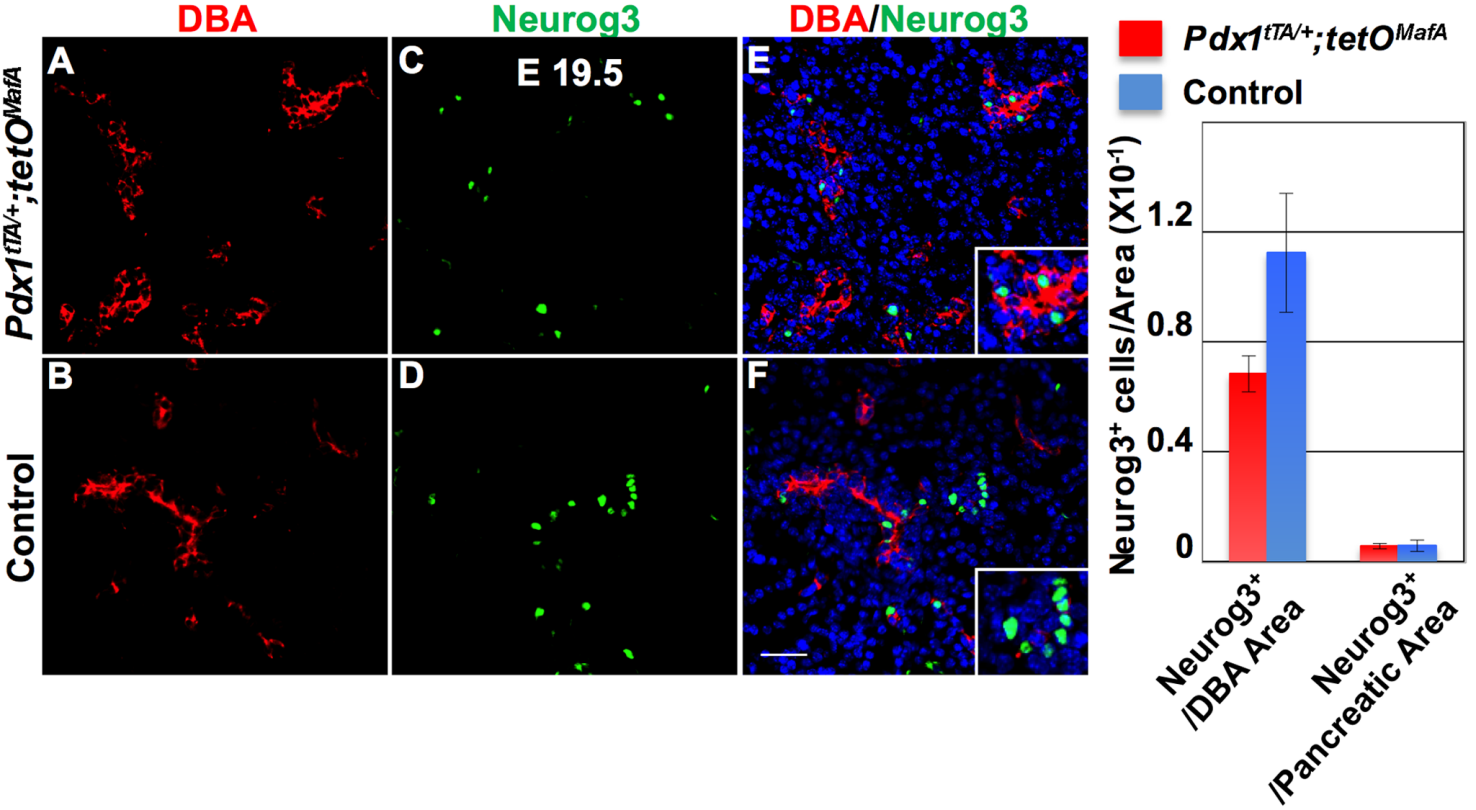
Quantification of Neurog3 expressing cells indicates compensatory increase in endocrine differentiation in bigenic mice. E19.5 pancreas of bigenic mice shows an increase in DBA^+^ cells but Neurog3^+^ cells (**ACE**) were comparable to controls (**BDF**). This equal Neurog3^+^ cell numbers is in contrast to the rare Neurog3^+^ cells seen in bigenics at E15.5 and E17.5 [17]. Quantification of Neurog3-expressing cells shows that the number of Neurog3-expressing cells normalized the pancreatic area for each section (**G**) are comparable in both bigenic and control E19.5 pancreas but showed a trend to being reduced in bigenics when normalized to the DBA^+^ area. DAPI (blue). Mean ± s.e.m. n = 3 Bar: 100 μm.

We previously showed that MafA^Myc^ expression in the Pdx1^+^ cells reduced the number of Neurog3^+^ endocrine progenitors at E15.5 and E17.5 [17]. However, here we observed that E17.5 bigenic tubular epithelium had increased proliferation (Fig 6) and "progenitor" potential (Fig 7) that was likely responsible for the increased endocrine cells at birth (Fig 5). The increased proliferation of DBA^+^ cells at E17.5 in bigenic pancreas (Fig 6) resulted in a tendency to increase DBA^+^ area in E19.5 bigenic pancreas compared to control (8.4+/-1.24% vs. 5.3+/-0.98%; p = 0.09) (Fig 9A and 9B). Furthermore, unlike the marked reduction in Neurog3^+^ cells in E15.5 and E17.5 bigenic pancreas [17], E19.5 bigenic pancreas had large numbers of Neurog3^+^ cells (Fig 9C). Quantification of Neurog3^+^ cells/DBA^+^ area (bigenic vs control: 6.8± 0.6 vs 11.2± 2.2 X10^-2^ cells/ μm^2^ of DBA area) or the total number of Neurog3^+^ cells to the pancreatic area at E19.5 (bigenic vs control: 0.6± 0.10 vs 0.6± 0.20 X10^-2^ cells/ μm^2^ of pancreatic area) showed comparable proportion of Neurog3 cells in control and bigenic pancreases. These data support that the increased endocrine cells at birth in bigenic pancreas resulted from increased endocrine specification and neogenesis in bigenic pancreas after E17.5 rather than enhancing proliferation of a few endocrine cells present at E17.5. Together these results demonstrate the ability of late-embryonic tubular epithelium to respond to the reduction in 1°MPC as well as pancreatic and endocrine mass, by enhancing their proliferation and increasing neogenesis of β-cells towards the end of gestation.

## Discussion

In our previous study [17] we reported that MafA transgene expression in Pdx1^+^ 1°MPC cells prevented proliferation of these cells resulting in reduced pancreatic weight and a marked decrease in endocrine cells at E17.5. Here we report that despite the reduction in pancreatic and endocrine mass during the secondary transition, tubular epithelial cells in the E17.5 *Pdx1* ^*tTA/+*^ *;tetO* ^*MafA*^ bigenic pancreas retained a significant compensatory ability to increase their proliferation and differentiation. This compensation resulted in a remarkable increase between E17.5 and P1 of both pancreatic size and number of endocrine cells. We show that this increase resulted primarily from enhanced proliferation of DBA^+^ tubular epithelial cells that expressed Glut2, a marker of embryonic progenitors [23] and not due to increased proliferation of endocrine cells present at E17.5. Together our data demonstrate that enforced expression of MafA in the 1°MPC inhibits their competency to specify endocrine progenitors only until E17.5 and that endocrine progenitors specified after E17.5 are either not dependent on such competency of 1°MPC or that a distinct pool of endocrine progenitors is specified after E17.5 in response to compensatory proliferation of tubular epithelium.

### Late embryonic tubular epithelium retains the capacity to specify all endocrine cell types

**Fig 10:**
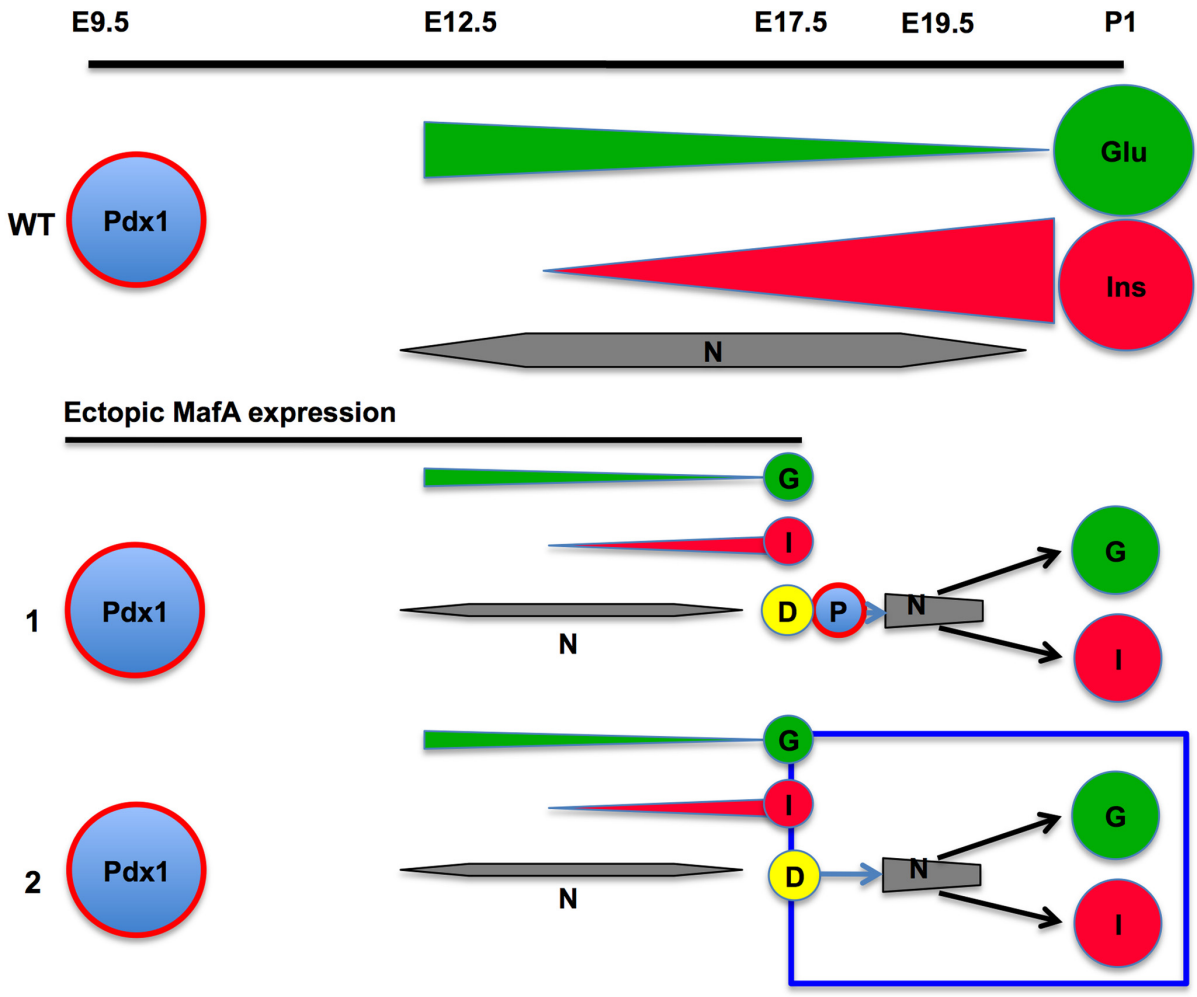
A schematic model depicting differential regulation of endocrine progenitors in control and bigenic pancreas. In control wild type (WT) pancreas (upper panel) Pdx1^+^ 1°MPC give rise to endocrine (Neurog3, N) progenitors that preferentially differentiate into α-cells during earlier stages of development and into β-cells at later. In lower panels the expression of MafA in 1°MPC prevents their expansion and differentiation [17] with few Neurog3^+^ or hormone^+^ cells at E17.5. However after release of this repression at E17.5, the hormone ^+^ cells increased by P1 even though there was no enhanced proliferation of the hormone^+^ cells at E17.5 or E19.5 as compared to controls but there were comparable numbers of Neurog3^+^ cells. Two scenarios are possible. In the first **(1)**, some of the 1°MPC progenitors (**P**) were retained in the tubular epithelium (D) and can resume differentiation and give rise to both α- and β-cells after E17.5 upon the release of MafA repression. In second **(2)**, 1°MPC are not retained in the bigenic tubular epithelium, but the E17.5 tubular epithelium itself (D) has the potential to differentiate into endocrine progenitors with competency to give rise to both α- and β-cells. Our results as presented in the **Fig 8** support the second scenario.

In bigenic mice there was a significant increase in the number of endocrine cells, increasing from occasional hormone^+^ cells seen at E17.5 to large clusters of hormone^+^ cells at birth (Fig 5). Since insulin^+^ cells at E17.5 and E19.5 showed comparable proliferation in bigenic and control pancreases, the increased endocrine cells at birth in bigenic mice could not be accounted by enhanced proliferation of few endocrine cells present at E17.5 (Fig 6). In contrast, proliferation of DBA^+^ cells was significantly higher at both E17.5 and E19.5 in the bigenic pancreas than in controls (Fig 6). These observations suggest that neogenesis from tubular epithelium is primarily responsible for the increased endocrine cells in bigenic mice. Although several cell types in adults may transdifferentiate into β-cells [13, 25–27], our results are consistent with the role of tubular epithelium as the primary source of beta cells during embryonic development. Lineage-marked Sox9^+^ cells from the late embryonic tubular epithelium have a modest capacity to give rise to endocrine cells [11, 12]. E17.5 tubular epithelial cells of both bigenic and control mice expressed Sox9 (Fig 7), suggesting that some of these cells can specify endocrine cells. As the Sox9 expression was seen in both bigenic and control pancreases, it is unlikely that Sox9 expression alone can explain the increased endocrine cells between E17.5 and P0 in the bigenic pancreas. However, the presence of DBA^+^ cells with higher expression of the progenitor marker Glut2 [23] in E17.5 bigenic but not control pancreas (Fig 7) provides an explanation for the differential neogeneic capacity of late-stage bigenic tubular epithelium. The significant increase in the number of endocrine cells in bigenic pancreas from E17.5 to P0 convincingly demonstrates the ability of late-embryonic tubular epithelium to specify endocrine cells, at least in response to a reduction in 1°MPC. This raises the possibility that the observed modest neogeneic potential of late-embryonic tubular epithelium [11, 12] could be enhanced in response to some developmental abnormalities. The ability of late embryonic tubular epithelium to differentiate into all endocrine cell type was surprising. Furthermore, a significant increase in the numbers of α-cells in the bigenic pancreas between E17.5 and P1 (Fig 5) is contrary to the previous report of dramatic reduction in competence of progenitors to specify α-cells after E14.5 [28] (Fig 10, WT situation). Our data that the endocrine progenitors specified during embryonic development after E17.5 retain the competence to specify α-cells and are insensitive to MafA expression in 1°MPC suggest that these progenitors differ from those specified earlier in development. However, it is possible that the enforced expression of MafA^Myc^ in 1°MPC results in retaining some of the 1°MPC in E17.5 tubular epithelium (Fig 10, situation #1), and upon the loss of MafA's repressive effect these progenitors resume differentiation. It is possible that such progenitors in the E17.5 tubular epithelium retain the competency of E12.5-E14.5 progenitors and specify α-cells even after E17.5. However, our BrdU retention study (Fig 8) showed that BrdU-labeled 1°MPC were not preferentially retained in the E17.5 bigenic tubular epithelium. Hence, we propose (Fig 10, situation #2) that endocrine progenitors specified after E17.5 are either not controlled by the competency of 1°MPC and can give rise to both α- and β-cells, or that a distinct pool of endocrine progenitors is specified after E17.5 in response to compensatory proliferation of tubular epithelial cells with a potential to give rise to both α- and β-cells.

These alternate possibilities may also impact the interpretation of our recent study where we observed that the enforced expression of MafA in endocrine progenitors blocked their differentiation into hormone-expressing cells at a stage after the progenitors committed to a hormonal fate [29]. The conclusion that a block in differentiation occurred after commitment of cell fate was based on the study that used *pdx1* promoter to drive Neurog3 expression in *Neurog3* knockout mice and showed the potential of endocrine progenitors to differentiate into α-cells diminishes after E14.5 [28]. It is important to note that the study by Johansson and colleagues [28] did not evaluate the potential of late-stage endocrine progenitors to differentiate into α-cells. Hence, in light of our present study, we suggest that until it can be confirmed whether the late-stage endocrine progenitors do or do not differentiate into α-cells, the question of whether the enforced expression of MafA in endocrine progenitors blocks their differentiation before or after they commit to a specific hormonal fate should remain open.

### Specification of endocrine progenitors is regulated at the level of 1°MPC

Similar to our results of inhibiting proliferation of pancreatic progenitors [17], diphtheria toxin-A (DTA) chain-dependent ablation of pancreatic progenitors caused a dramatic reduction in pancreatic size and resulted in a more drastic phenotype [5]. The induction of DTA in Pdx1^+^ cells only until E10.5 [5] resulted in a modest reduction in pancreatic size but a differential reduction in endocrine number at E18.5 comparable to what we saw at E17.5 (Figs 2 and 3)[17]. Thus both our results and those of Stanger [5] support a role of 1°MPC in regulating the subsequent differentiation of endocrine cells. Our observation of reduced endocrine transcription factors-expressing cells at E17.5 (Fig 3 and S1 Fig) suggests that enforced expression of MafA^Myc^ inhibits the endocrine differentiation program. The reduced number of Neurog3^+^ cells at E15.5 [17] further supports the conclusion that MafA^Myc^ expression in 1°MPC blocks the specification of endocrine progenitors during secondary transition rather than preventing differentiation of endocrine progenitors into endocrine cells.

The induction of transient Neurog3^Hi^-expressing cells in tubular epithelium marks the cells that will acquire endocrine fate. These cells migrate out of the tubular epithelium, reduce their Neurog3 expression, and differentiate into hormone^+^ cells [3, 30–33]. In the absence of Neurog3 [31] a lack of lateral inhibition of adjacent cells resulted in endocrine specification throughout the tubular epithelium (as determined by the expression from *Neurog3* promoter) and the lack of migration of these marked cells out of the tubular epithelium caused enlarged/abnormal tubular structures. The absence of similarly enlarged/abnormal tubular structures at E17.5 in the bigenic pancreas (Fig 2) supports our conclusion that MafA^Myc^ expression inhibits the specification of endocrine progenitors and not their differentiation. Together these observations suggest that preventing proliferation of 1°MPC results in the reduction in pancreatic progenitors, and even a greater reduction in endocrine progenitors than that can be explained by the reduction in the pancreatic progenitors alone. Our results also demonstrate that the ability to induce *Neurog3* expression during the secondary transition is committed in the 1°MPC before E12.5 and that specification of endocrine progenitors from the tubular epithelium is likely regulated by distinct mechanisms during and after the secondary transition. However, it is possible that transgene misexpression in the 1°MPC only limits the ability of tubular epithelial cells to specify endocrine progenitors until the secondary transition (here by E17.5) and after that these cells regain their differentiation potential.

### Reduced numbers of 1°MPC trigger a compensatory response from late embryonic tubular epithelium

Between E17.5 and P0 the mass of the bigenic pancreas increased (Fig 4) but was still less than controls. Stanger and colleagues [5] reported that unlike the liver the pancreas was unable to compensate for a reduced progenitor pool and did not show a catch-up growth even after birth. Hence, we expected that pancreatic growth between E17.5 and P0 in bigenic (Figs 4 and 5) would be proportional to that in control. However, we observed a relatively larger increase in insulin (3.8-fold vs. 1.3 fold), glucagon (3.0-fold vs. 1.1 fold) and pancreatic areas (4.8-fold vs. 2.0 fold) between E17.5 and P0 in bigenic mice than in controls (Fig 5). In response to the reduced pancreatic and endocrine mass, there was a significant increase in proliferation of E17.5 and E19.5 bigenic late-embryonic tubular epithelium (Fig 6). Furthermore, in contrast with the dramatic reduction in Neurog3^+^ cells seen in E15.5 bigenic pancreas [17], the number of Neurog3^+^ cells dramatically increased by E19.5 (Fig 9G). This increase in Neurog3^+^ cells led to comparable number of endocrine progenitors/pancreatic area in bigenic and control pancreases (Fig 9G). Similarly, the proportion of Neurog3^+^ / DBA^+^ cells did not differ between the bigenics and controls (Fig 9). These observations indicate that the efficiency of individual late-gestational DBA^+^ cells to differentiate into Neurog3^+^ cells did not differ between the bigenic and control pancreases and that the increased proliferation of tubular epithelial cells was likely the key regulatory step responsible for the increased endocrine mass in bigenic pancreas at P0. As we saw a dramatic increase in the number of both Neurog3^+^ cells and endocrine cells after E17.5, we suggest that this induction of Neurog3^+^ cells was responsible for most, if not all, endocrine cells present at birth. However, as stated before, this increase was not sufficient to normalize the blood glucose levels (Fig 5N).

These results show for the first time the presence of a mechanism that regulates the amount of endocrine progenitors present at the late embryonic stage. However, at present it is not possible to resolve whether the compensatory response requires a reduction in pancreatic/endocrine mass following the loss of 1°MPC or it is a normal mechanism that regulates the relative proportion of endocrine progenitors in late embryonic stage pancreas. It is important to state that this capacity of the tubular epithelium to compensate for the loss of pancreatic progenitors by increasing both pancreatic and endocrine cells is seen at an age that was not examined by Stanger and colleagues [5]. We cannot rule out that the differences between our results and those reported by Stanger and colleagues [5] may be due to different strategies used in these studies to reduce progenitor population. Forced MafA^Myc^ expression prevented proliferation of pancreatic progenitors [17], while DTA expression resulted in ablation of progenitors [5]. Examining proliferation and differentiation of late-embryonic tubular epithelium from mice with DTA-mediated ablation of pancreatic progenitors may help resolve this issue.

## Conclusions

Our results reveal that forced MafA expression in 1°MPCs either 1) inhibits their competency to specify endocrine progenitors only until E17.5, 2) two distinct pools of endocrine progenitors are specified during normal pancreatic development, or 3) the MafA-unresponsive endocrine progenitor pool is specified upon compensatory proliferation of tubular epithelium from late gestational age. For the second and third possibilities to be true, the specification of endocrine progenitor pool before E17.5 must depend on 1°MPC and be sensitive to enforced MafA expression, while the specification of a second progenitor pool after E17.5 should be regulated by a distinct mechanism. Examination of endocrine progenitors formed before and after E17.5 for their potential to differentiate into mature endocrine/β-cells and evaluation of the role of 1°MPC in the specification of both endocrine progenitor populations, will significantly enhance our understanding of endocrine differentiation and formation of mature β-cells. Additionally, our results demonstrate that the embryonic pancreas contains a modest compensatory response to a reduction in pancreatic size and endocrine mass. However, the compensatory increase in the endocrine cells at birth in our experimental system was not sufficient to prevent hyperglycemia. We suggest that additional studies are needed to determine if we can enhance this compensatory increase in tubular epithelial proliferation to normalize blood glucose levels and to decipher if the increased proliferation of these cells resulted only from the loss of 1°MPC or required the loss of pancreatic and/or endocrine mass. The presence of such a compensatory response in the embryonic (and likely adult) pancreas suggests that enhancing proliferation of tubular/ductal epithelium represents a key pathway to increase endocrine differentiation and beta cell mass.

## Supporting Information

S1 Fig Endocrine precursor cells are located within or next to branching duct structure in *Pdx1* ^*tTA/+*^ *;tetO* ^*MafA*^ embryos at E17.5. At E17.5 *Pdx1* ^*tTA/+*^ *;tetO* ^*MafA*^ pancreas had cells within or next to a branching duct structures (dashed lines) that expressed Neurog3 (green, **A**), Nkx2.2 (green, **BC**), Nkx6.1 (green, **DE**), Pax6 (green, **F**) and Isl1 (green, GH) indicating that these cells were endocrine precursor cells. Insulin expression (red, **H**) was rarely observed, but a few cells expressing insulin were seen along the lining of duct epithelium. DAPI (blue). Bar: 20 μm.
(PDF)
