# Supplementary material for: Compensatory Response by Late Embryonic Tubular Epithelium to the Reduction in Pancreatic Progenitors
Source: PLoS One. 2015 Nov 5;10(11):e0142286. doi: 10.1371/journal.pone.0142286 (PMC4635002; doi:10.1371/journal.pone.0142286)
Supplement: S1 Fig — At E17.5 Pdx1 tTA/+ ;tetO MafA pancreas had cells within or next to a branching duct structures (dashed lines) that expressed Neurog3 (green, A), Nkx2.2 (green, BC), Nkx6.1 (green, DE), Pax6 (green, F) and Isl1 (green, GH) indicating that these cells were endocrine precursor cells. Insulin expression (red, H) was rarely observed, but a few cells expressing insulin were seen along the lining of duct epithelium. DAPI (blue). Bar: 20 μm. (PDF) [file pone.0142286.s001.pdf]

S1 Fig. Endocrine precursor cells are located within or next to branching duct structure in *Pdx1*$^{tTA/+}$*;tetO*^{MafA} embryos at E17.5. At E17.5 *Pdx1*$^{tTA/+}$*;tetO*^{MafA} pancreas had cells within or next to a branching structures (dashed lines) that expressed Neurog3 (green, **A**), Nkx2.2 (green, **BC**), Nkx6.1 (green, **DE**), Pax6 (green, **F**) and Isl1 (green, **GH**) indicating that these cells were endocrine precursor cells. Insulin expression (red, **BCD**) was rarely observed, but a few cells expressing insulin were seen along the lining of duct epithelium. DAPI (blue). Bar: 20μm.

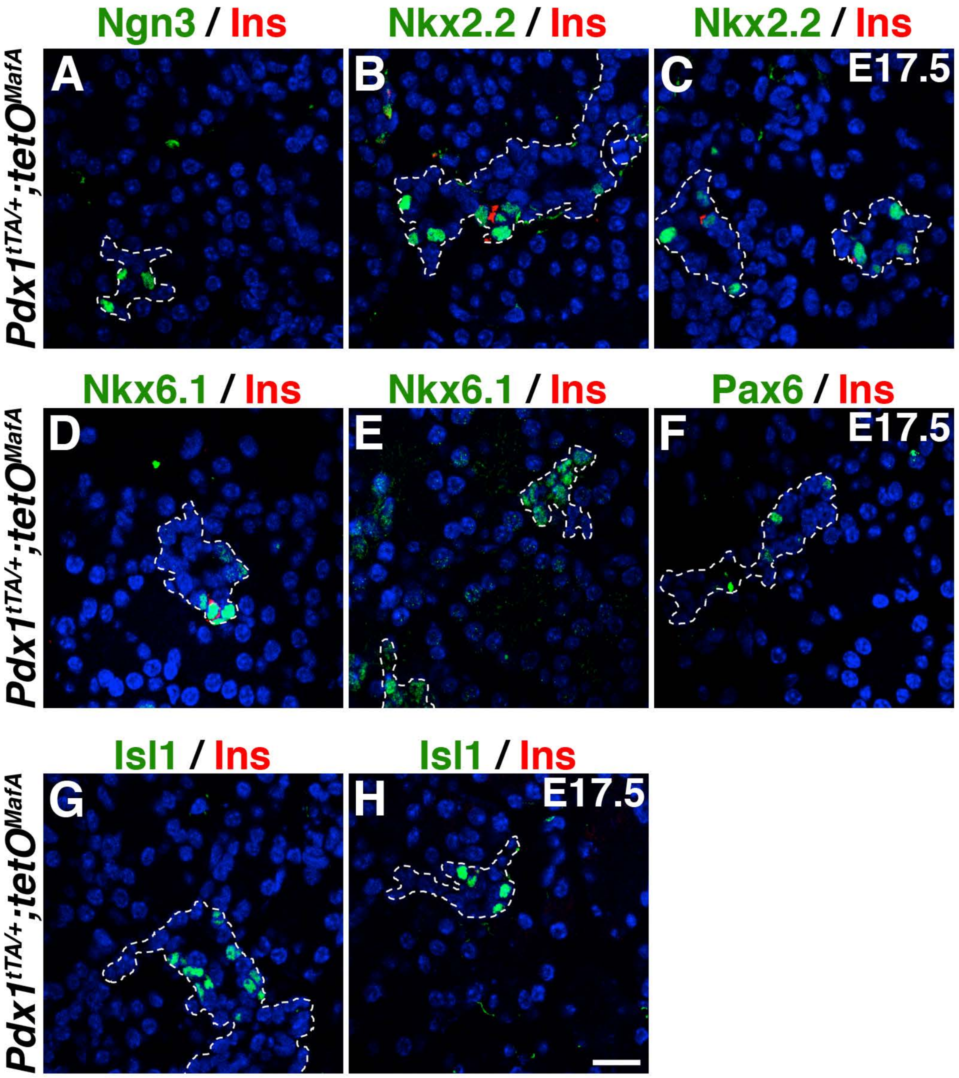
Ngn3 / Ins
Nkx2.2 / Ins
Nkx2.2 / Ins
Pdx1$^{tTA/+}$;tetO^{MafA}
A
B
C
E17.5
Nkx6.1 / Ins
Nkx6.1 / Ins
Pax6 / Ins
Pdx1$^{tTA/+}$;tetO^{MafA}
D
E
F
E17.5
Isl1 / Ins
Isl1 / Ins
Pdx1$^{tTA/+}$;tetO^{MafA}
G
H
E17.5

S1 Fig
